# Supplementary material for: Physical Activity, Nutritional Behaviours and Depressive Symptoms in Women with Hashimoto’s Disease
Source: Healthcare (Basel). 2025 Mar 13;13(6):620. doi: 10.3390/healthcare13060620 (PMC11942261; doi:10.3390/healthcare13060620)
Supplement: Supplementary file 1 [file healthcare-13-00620-s001.zip › healthcare-3476632-supplementary.pdf]

Questionnaire of Nutritional Behaviours for Individuals Hypothyroidism (QNB)

Author: Maria Gacek

In the table, various dietary behaviours are presented. Please indicate, as precisely as possible, to what extent the following statements apply to your usual dietary habits in relation to the symptoms of Hashimoto's disease. Please mark the choice that best describes your behaviour with an X in the appropriate column. Please, remember that there are no right or wrong answers. Thank you for your honest answers.

Respondent code: ………..…………………………………

Date: ……………………………………………..

**Behaviours**	**Definitely do not**	**Rather do not**	**Difficult to say** (neither do nor do not)	**Rather do**	**Definitely do**
I regularly consume products rich in iodine (including sea fish, seafood, iodized salt)					
I regularly consume foods rich in iron (including fish, meat, liver, legumes, green vegetables, nuts, whole grains)					
I regularly consume foods rich in zinc (including meat, eggs, oilseeds, legumes, garlic, onions, mushrooms)					
I regularly consume products rich in selenium (including Brazil nuts, sea fish, legumes, poultry, mushrooms, whole grains, yellow cheeses)					
I regularly consume products rich in vitamin D (including sea fish, cod liver oil, D3 supplements)					
I regularly consume products rich in vitamin A (including cheeses, egg yolks, oily fish, yellow, orange and red vegetables and fruits)					
I regularly consume products rich in omega 3 fatty acids (including sea fish, linseed oil, walnuts)					
I limit the consumption of cruciferous vegetables (including cabbage, brussels sprouts, kale, cauliflower, kohlrabi)					
I limit the consumption of soy and its products, especially processed ones					
I follow a gluten-free diet due to gluten intolerance					
I avoid dairy products due to casein intolerance					
I follow a lactose-free diet due to lactose intolerance					
I follow a weight loss diet due to excess body mass					
I follow a diet with a lot of fibre (including whole grains, bran, thick groats, vegetables) due to constipation					
I consume a meal (breakfast) 30-60 minutes after taking medication for					

hypothyroidism					
During breakfast (after taking thyroxine) I do not consume products rich in calcium (including milk, yellow cheese, almonds, fish with edible bones)					
I drink coffee or strong tea only after about two hours after taking the medication for hypothyroidism					
I take dietary supplements only a few hours after taking the medication for hypothyroidism					
I follow a paleo diet (I eat meat, fish, eggs, green vegetables, nuts, and I do not eat milk, grains, legumes, or processed products)					
I periodically use the autoimmune protocol (to alleviate the symptoms of hypothyroidism)					
I follow doctor's recommendations and I do the recommended tests					
